# Progress in the Development of CdZnTe Unipolar Detectors for Different Anode Geometries and Data Corrections

**DOI:** 10.3390/s130202447

**Published:** 2013-02-18

**Authors:** Qiushi Zhang, Congzhe Zhang, Yanye Lu, Kun Yang, Qiushi Ren

**Affiliations:** 1 Department of Biomedicine and Engineering, Peking University, No. 5, Yiheyuan Road, Beijing 100871, China; E-Mails: zhangqsh@pku.edu.cn (Q.Z.); becky.lyy@gmail.com (Y.L.); 2 Department of Control Technology and Instrument, Hebei University, No. 180, Wusi East Road, Baoding 071000, China; E-Mail: czhang015@gmail.com

**Keywords:** hole trapping, weighting potential, Frisch grid, coplanar grid, strip electrode, 3-D position sensing, Compton imaging

## Abstract

CdZnTe detectors have been under development for the past two decades, providing good stopping power for gamma rays, lightweight camera heads and improved energy resolution. However, the performance of this type of detector is limited primarily by incomplete charge collection problems resulting from charge carriers trapping. This paper is a review of the progress in the development of CdZnTe unipolar detectors with some data correction techniques for improving performance of the detectors. We will first briefly review the relevant theories. Thereafter, two aspects of the techniques for overcoming the hole trapping issue are summarized, including irradiation direction configuration and pulse shape correction methods. CdZnTe detectors of different geometries are discussed in detail, covering the principal of the electrode geometry design, the design and performance characteristics, some detector prototypes development and special correction techniques to improve the energy resolution. Finally, the state of art development of 3-D position sensing and Compton imaging technique are also discussed. Spectroscopic performance of CdZnTe semiconductor detector will be greatly improved even to approach the statistical limit on energy resolution with the combination of some of these techniques.

## 1. Introduction

**Figure 1.**
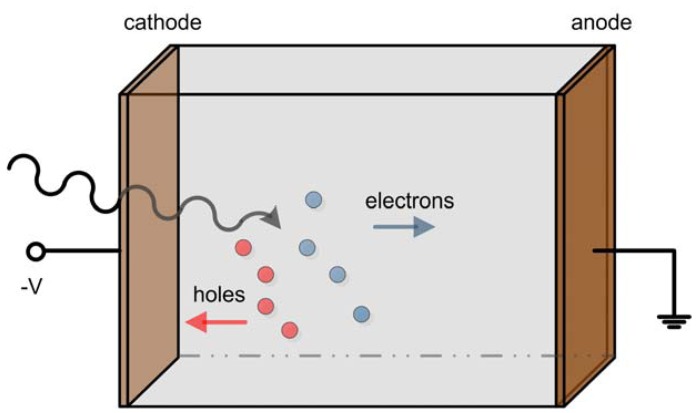
Planar configuration of a semiconductor detector. The cathode is applied with a negative voltage and anode grounded. Electron-hole pairs excited by gamma rays are swept by the bias voltage.

Semiconductor nuclear radiation detectors have experienced a rather rapid development in the last few decades. A major characteristic of this type of detector is the capability of converting γ-rays directly into electronic signals. In comparison to scintillators, semiconductor detectors avoid the random effects associated with scintillation light production, propagation and conversion to electrical signal in such a way that they represent the main alternative to scintillator-based single photon imaging systems. Compared to established use of Si and Ge, cadmium zinc telluride (CdZnTe) is the most promising material for radiation detectors with high atomic number (good stopping power), large band-gap (room-temperature operation), and the absence of significant polarization effects [1,2]. The incident gamma-ray interacts with the semiconductor and excites electron-hole pairs, that are proportional to the deposited energy and drifts apart under the applied electric field. Electrons drift towards the anode and holes drift towards the cathode (Figure 1). Charge signal is induced on the electrodes of the detector by the moving charge carriers. It is due to the direct conversion from the energy deposition by gamma-ray interaction to electric signal that semiconductor detector can easily achieve high energy resolution and spatial resolution [3]. In practice, CdZnTe materials exhibit varying degrees of charge carrier trapping, which in fact is the dominant problem that has limited their energy resolution. Our group at the Molecular Imaging Lab of Peking University is developing micro single-photon emission computed tomography (SPECT) for small animal imaging. This paper reviews the techniques of overcoming hole trapping problems for CdZnTe detector, including particular irradiation configuration of the same, electronic methods to distinguish events from a large contribution of the holes and the various electrode designs. We also review the state of art development of 3-D position sensing and Compton imaging techniques using CZT detectors.

## 2. Theoretical Principles

The collection efficiency of charge carriers is a crucial property that affects the energy resolution of semiconductor detectors. This efficiency is always reduced by charge carriers trapping that results from crystal defects and the poor charge transport properties of charge carriers. For example, grain boundaries that are generated during crystal growth can seriously trap charge carriers [4]. It has been shown that spatial non-uniformity of semiconductor materials will cause a loss of energy resolution [5,6]. In addition, the mean drift length of electrons is typically of the order 1 cm while this length of holes is much lower than that of electrons with values around 0.1 cm under typical electric fields of 1,000 V/cm. With poor charge collection, the charge signal induced on the electrode is reduced, which is more pronounced for events that occur further away from the collecting electrode. This is how the position-dependent signal variation is produced.

**Figure 2.**
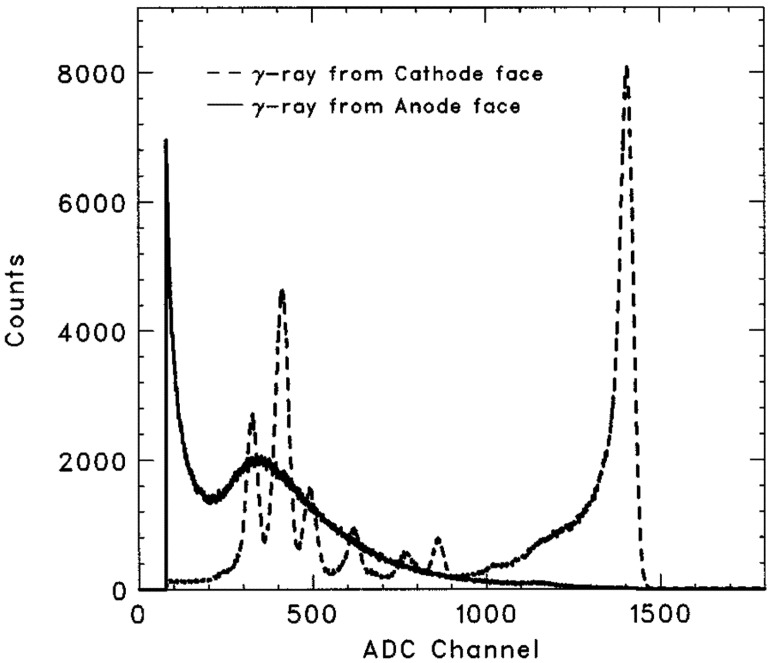
A typical ^241^Am spectrum obtained with 4 × 4 × 2 mm^3^ CZT detector, which is irradiated from the cathode side (dashed line) and anode side (solid line). The photopeak can be resolved if gamma rays are irradiated from the cathode side but not from the anode side [7] (©IEEE, 2001).

Figure 2 shows two typical spectra obtained with a CdZnTe planar detector when irradiated from anode and cathode side, respectively. The anode-irradiating spectrum has no peak because most of the holes are trapped due to a long drift distance to the cathode. However, the peak can be clearly resolved on the cathode-irradiating spectrum. Since the amplitude of the induced charge signal depends on the depth of interaction (DOI), the cathode-irradiating spectrum still shows a tailing.

### 2.1. Shockley-Ramo Theory

As already stated, the charge carriers that are generated by γ-photon energy deposit drift towards the corresponding electrodes. Shockley and Ramo proposed a method in 1940s to calculate the induced charge by introducing a concept of “weighting potential” [8–10]. The charge variance (*ΔQ_L_*) that is achieved by a moving charge *q* from interaction position *x_i_* to *x_f_* and induced on the electrode (*L*), can be calculated according to Equation (1):

(1) $$\Delta Q_{L}=\int_{x_{i}}^{x_{f}}qE_{0}\cdot dx=-q[\varphi_{0}(x_{f})-\varphi_{0}(x_{i})].$$

where *x_i_* to *x_f_* is the initial and final position of *q*, and *E_0_* and *φ_0_* correspond to the weighting electric field and weighting potential respectively. Weighting potential (electric field) is defined as the potential (electric field) that would exist in the detector when the collecting electrode is biased at unit potential and all other electrodes are held grounded. It does not really exist inside the detector but is only for calculation convenience. Note that the induced charge is independent of the applied bias voltage on the electrodes. That voltage only determines the trajectories of charge carriers.

### 2.2. Static Charge Analysis and Capacitance Coupling Method

**Figure 3.**
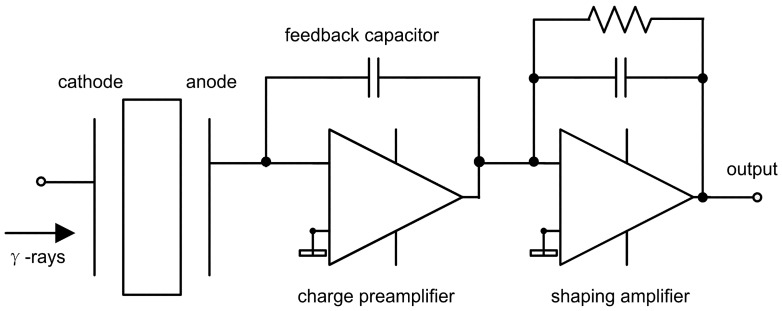
Typical amplification circuit of a semiconductor planar detector. The total charge on the feedback capacitor consists of two parts: free electrons that drift to the anode and the charges induced by trapped electrons and holes within the detector. Positive charges induced on the anode will add electrons and negative charges will reduce electrons on the feedback capacitor. The amplitude of output signal is proportional to the charges that are generated on the feedback capacitor.

Another approach to calculate the output charge on the electrode, called “static charge analysis and capacitance coupling method”, was introduced by Lingren and Butler [11]. What is shown in Figure 3 is a typical amplification circuit model. The total amount of charge (*Q_total_*) that is generated on the feedback capacitor consists of two parts—free electrons that are collected directly by the electrodes and the charges induced by trapped carriers within the detector (Equation (2)):

(2) $$Q_{\text{total}}=Q_{hi}+Q_{ei}+Q_{ef}$$

where *Q_hi_* and *Q_ei_* are charges that are induced by trapped holes and electrons respectively on the feedback capacitor and *Q_ef_* is the free electrons collected on the anode and conducted onto the feedback capacitor. Suppose that the free charge carriers that are created by photons absorbtion are +*Q_0_* (holes) and -*Q_0_* (electrons) and that the interaction position is at a distance of *x_0_* from the cathode. When these carriers drift towards the respective electrodes with initial velocities of *μ_hE_* (holes) and *μ_eE_* (electrons), the number of them is decreased due to charge trapping within the detector. Here, *E* is the applied uniform electric field, *μ_h_* the hole mobility and *μ_e_* the electrons mobility. Moreover, there is an assumption that a loss of charge caused by charge trapping proceeds exponentially with time (Equations (3) and (4)) and detrapping is ignored. Thus the amount of free charge of electrons (*Q_ef_*) and holes (*Q_hf_*) with the change of the position *x* in the drift path, can be then obtained as:

(3) $$Q_{e\text{f}}(x)=-Q_{0}e^{-(x-x_{0})/\lambda_{e}}(x\geq x_{0})$$

and:

(4) $$Q_{h\text{f}}(x)=+Q_{0}e^{(x-x_{0})/\lambda_{h}}(x\leq x_{0})$$

The amount of charge, which is induced on an anode by infinitesimal charges *dQ_ef_(x)* and d*Q_hf_(x)*, is equal to the infinitesimal charge multiplied by a weighting factor. This weighting factor is a ratio of the capacitance from the interaction point to the collecting anode to the total capacitance from that point to all electrodes. Thus the infinitesimal induced charges (*dQ_ei_(x)* and *dQ_hi_(x)*) on the anode can be given as:

(5) $$dQ_{ei}(x)=-\frac{C_{a}(x)}{C_{t}(x)}\left|dQ_{ef}(x)\right|$$

and:

(6) $$dQ_{hi}(x)=-\frac{C_{a}(x)}{C_{t}(x)}\left|dQ_{hf}(x)\right|$$

where *C_a_(x)* is the capacitance from the interaction point *x* to the collecting anode, *C_t_(x)* is the total capacitance from that point to all electrodes. Here we use an example of a planar detector to verify Lingren's method. For a planar detector, the weighting factor *C_a_(x)/ C_t_(x)* is equal to *x/L* [12], where *L* is the detector's thickness. Then the total induced charge (*Q_i_*) on anode is obtained as:

(7) $$\begin{array}{l} Q_{i}(x)=\int_{0}^{x_{0}}\frac{x}{L}dQ_{h}(x)+\int_{x_{0}}^{L}\frac{x}{L}dQ_{e}(x)\\ =\frac{Q_{0}}{\lambda_{h}}\int_{0}^{x_{0}}e^{(x-x_{0})/\lambda_{h}}\frac{x}{L}dx-\frac{Q_{0}}{\lambda_{e}}\int_{x_{0}}^{L}e^{-(x-x_{0})/\lambda_{e}}\frac{x}{L}dx\\ =-\frac{Q_{0}}{L}[\lambda_{e}(1-e^{-(L-x_{0})/\lambda e})+\lambda_{h}(1-e^{-x_{0}/\lambda_{h}})]+Q_{0}e^{-(L-x_{0})/\lambda e} \end{array}$$

*Q_total_* can be obtained by pluging *Q_ef_(L)* (Equation (3)) and *Q_i_(x)* (Equation (7)) into Equation (2). The result (Equation (8)) is identical to that obtained by the Hecht equation, thus, verifying Lingren's method:

(8) $$Q_{\text{total}}=-\frac{Q_{0}}{L}[\lambda_{e}(1-e^{-(L-x_{0})/\lambda e})+\lambda_{h}(1-e^{-x_{0}/\lambda_{h}})]$$

## 3. Techniques to Reduce Hole Trapping

Energy resolution is one of the main performance parameters for gamma ray detectors. As seen from Figure 2, the low energy branch in a typical spectrum always presents as a tailing. This tailing primarily results from hole trapping and degrade energy resolution; it is a necessary requirement to overcome hole trapping in order to improve detector performance. Three ways to achieve this: configuring irradiation direction, distinguishing events with a large contribution of holes using electronic methods and minimizing the sensitivity to holes through detector geometry design.

### 3.1. Irradiation Direction Configuration

As mentioned previously, hole trapping limits detector performance. This is because a long tail is produced in the measured spectrum due to incomplete charge collection. It was observed that irradiation from cathode side can contribute to reducing this effect [7] because the counts of events that are excited near the cathode are increased, thus minimizing the probability of hole trapping. This irradiation configuration is more effective for low energy rays in thin detectors. Another advantage of using the cathode-irradiating configuration is the uniform trigger rate effect, since using a cathode electronic signal to trigger the acquisition of electrode pulse heights can lead to much more uniform event acquisition response than using anode pixel signal [13].

Another irradiation configuration, in which the irradiation direction is orthogonal to the applied electric field, was being considered as a way of overcoming the compromise between good spectroscopy and acceptable detection efficiency [14,15]. In configuration of this type, the interaction position information can be obtained experimentally due to the fact that the photopeak centroid value is correlated with interaction position. And different detector thicknesses can be chosen in order to get the required detection efficiency. Nevertheless, a tradeoff between the required energy resolution and irradiated area should be considered. This method is particularly useful for developing detectors when high detection efficiency is required.

### 3.2. Pulse Shape Correction

Electronic methods have also been used to improve the spectrometric performance of CdZnTe detectors, such as pulse shape discrimination (PSD) [16–19] and pulse rise-time compensation (PRC) [17,20]. Both of these techniques are implemented in combination with hardware and software. PSD method was developed to distinguish events with a large contribution from hole trapping since events with a large degree of hole trapping always present an output pulse with a long rise-time. These events can be eliminated in such a way that high spectral resolution can be achieved at the cost of rejecting a fraction of the pulses, thus resulting in a drastic loss in detection efficiency. Alternatively, PRC method was developed to allow for reducing the loss of efficiency while obtaining energy resolution improvement. The aim of PRC is to compensate pulse amplitude of those events with severe hole trapping according to pulse shape characteristic. Different approaches were reported to realize PRC through different bi-parametric spectrums (BPS), such as “pulse amplitude & pulse rise-time” spectrum [17] as well as “fast/slow ratio & slow signal” spectrum [20]. BPS is a useful analysis tool and has a real advantage in spectrum correction, e.g., scattering rejection [2,21].

A novel algorithm, used for rejecting incomplete charge collection (ICC) events in CdZnTe detectors, was proposed by Bolotnikov *et al.* [22]. This method is based on a BPS that is reflected in R-T function and features for reducing the Compton continuum in the energy spectrum. The R-T function is a unique function of the detector correlating the ratio of cathode to anode signal and drift times measured for each detected event. Some events falling out of a curve that represents the correlation function were regarded as ICC events and thus rejected. In this way, the Compton continuum and the low-energy tail of the energy spectrum can be greatly reduced without affecting the photopeak efficiency significantly. This algorithm is much more effective in correcting virtual Frisch-grid CdZnTe detectors and can also be employed practically for any single charge sensing type detectors.

## 4. Unipolar Detectors of Different Anode Geometries

To some extent, both of the methods mentioned in Sections 3.1 and 3.2 can reduce hole trapping effect on the charges that are collected by electrodes; these methods still remaining insufficient to obtain a good quality energy resolution. In addition, drastic losses in detection efficiency are caused with a limited improvement in energy resolution, especially with thicker detectors. Therefore, the approaches mentioned above could be adjunct methods to obtain better energy resolution or furthermore could be used in certain occasions where efficiency is a less important parameter. Unipolar detector designs, however, have been developed to overcome the deleterious effects of hole trapping problem. The most effective and successful prototype models, listed and mentioned below, include the Frisch-grid device, pixelate detectors, coplanar grid detectors, hemispherical electrodes and strip detectors.

### 4.1. Frisch Grid Device

#### Frisch grid effect

**Figure 4.**
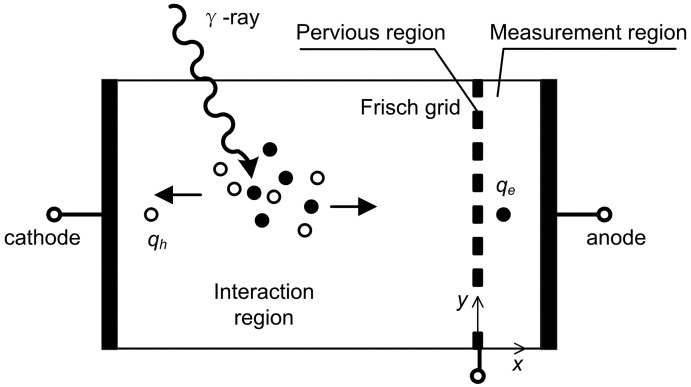
Schematic of the basic configuration for the Frisch grid detector. Three regions that are separated by the grid can be seen. Charge carrier e.g., *q_e_* which drifts into the measurement region will induce charges on the anode, while *q_h_* in the will not.

**Figure 5.**
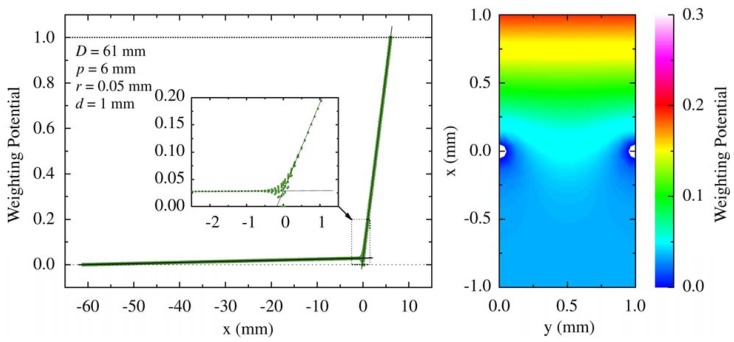
Weighting potential distribution of the Frisch grid detector. X- and Y-coordinate are indicated in Figure 4. **left:** caculated weighting potential projected on the X-coordinate. D (*p*) are the distance between the cathode (anode) and the grid. *d* is the distance between the grid elements and *r* is the half length of the element. As expected, weighting potential value for x < 0 is nearly zero and abruptly changes for x > 0. A slight y-dependence of weighting potential is shown in the inset map that zooms into the origin of X-coordinate. **Right:** 2-D map of weighting potential around the Frisch grid (X: −1 to +1, Y: 0 to 1.0) [25] (Image courtesy of Elsevier).

Frisch-grid-based design that was introduced by Frisch [23] was originally used for gas-filled ion chambers. The aim of this design was to make the induced charge on the collecting electrode insensitive to the carriers with lower mobility. Thus the induced charge can be measureed primarily from the carriers with higher mobility. A general configuration of a Frisch grid ion chamber is shown in Figure 4. A Frisch grid is placed in the vicinity of the anode so that three distinctive regions are formed. The region between cathode and Frisch grid is called interaction region where most gamma rays interact, and the region between anode and Frisch grid is the measurement region where the induced charges are measured. The pervious region, where charge carriers pass through, is underneath the Frisch grid. According to Shockley-Ramo theory, the weighting potential (Figure 5) can be obtained by setting the potential of the anode to 1 and the cathode and Frisch grid to 0 [24]. From this viewpoint, the weighting potential in the interaction region is invariable so that charge motion in this region has no contribution to the induced charge on the anode. As a result, the detector is primarily sensitive to the electron charge carriers passing through the measurement region.

#### Frisch grid detectors

**Figure 6.**
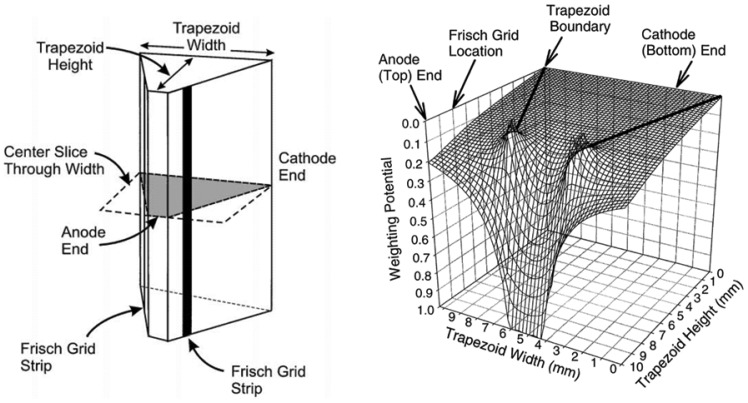
**Left:** 3-D geometric diagram of trapezoid prism detector. The anode has a smaller area with two parallel strips at along sides as the Frisch grids. The trapezoid height is often several times longer than the distance between the Frisch grid and the anode so that a much smaller volume of measurement region is generated. A cross-section cut through the width in the device center is shown; **Right:** weighting potential distribution of the cross-section as shown in the left diagram [28] (Image courtesy of Elsevier).

The most studied structures for such semiconductor detectors include the Frisch strip detector [24,26], trapezoid prism detector [27,28] and insulated Frisch ring detector [29–34]. The first single model was built by McGregor *et al.* [24,26] with two parallel metal strip electrodes on opposite faces of the device. The metal strips, similar to Frisch grid, have a small width and are placed near the anode so that the measurement region is considerably smaller than the interaction region. In addition, a negative voltage can be applied to both of the two side strips to assist electrons in drifting towards the anode. Energy resolution of 5.91% FWHM at 662 keV gamma rays was obtained with this configuration. This result is less-than-ideal; nevertheless, it was a great improvement in energy resolution in comparison with planar detectors. It was also observed that energy resolution results were correlated with width-to-length ratio of the strip. Another version of the Frisch grid device, called the trapezoid prism detector (Figure 6), was developed by combining geometric weighting effect, small pixel effect (mentioned in Section 3.3.2) and Frisch grid effect [27]. Main characteristic of this device is its geometric shape design, e.g., a larger volume of interaction region. This allows the fraction of events occurring in the interaction region to become dramatically increased. Accordingly, the gamma ray sensitivity in this region is enhanced. The energy resolution achieved with this device is 2.68% at FWHM for 662 keV gamma rays without any correction or processing, and that performance exceeds the Frisch strip detector by an enormous amount. The additional study have also demonstrated the soundness of this design [28].

**Figure 7.**
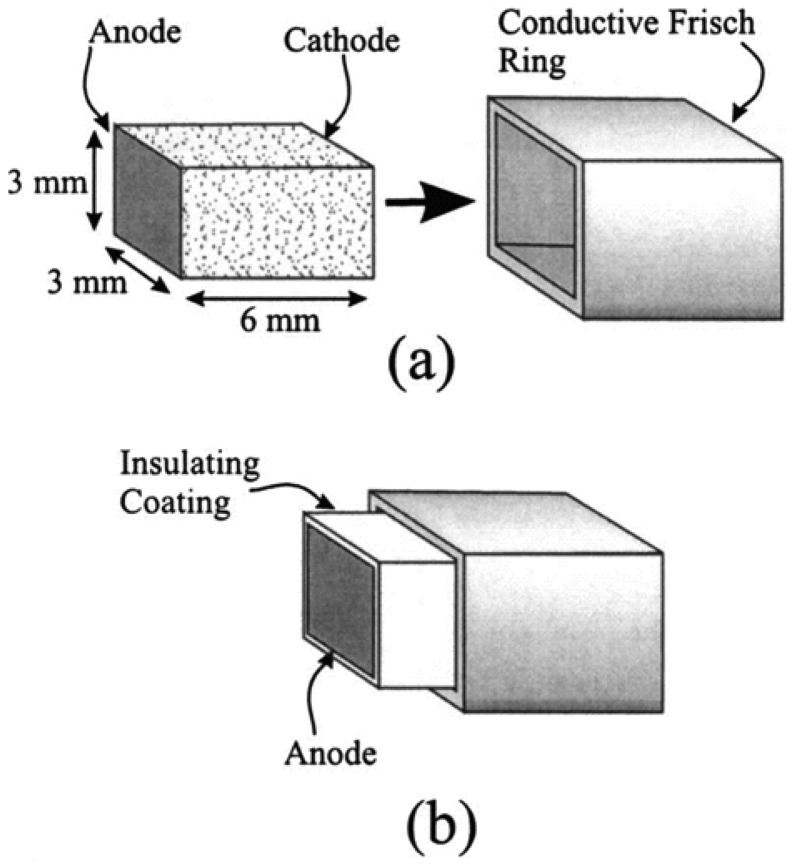
The capacitive Frisch grid device and its components: a bar-shaped detector, an insulating coating and a conductive Frisch ring. (**a**) The detector bar with Teflon coated outside is non-contacted with the Frisch ring. (**b**) When the detector is inserted into the Frisch ring, the special weighting potential near the anode is generated and thus effectively eliminating the induction from charge motion in the region extending from the ring edge to the cathode [29] (Image courtesy of American Institute of Physics).

Both of the two structures mentioned above suffer from severe surface leakage currents between the grid and anode, especially under conditions of higher applied voltage. This problem is definitely a limitation for detectors when trying to gain better performance. The capacitive Frisch grid detector (or “insulated Frisch ring detector”, Figure 7) designed by McGregor and colleagues [29,30,33] was based on the concept of screening effect [34].

This design consists of a bar-shape detector and a conductive ring with a thin layer of dielectric material between them, which greatly reduces the leakage current between the grid and anode due to the non-contacting effect. Excellent results of energy resolution were obtained of 1.7% FWHM at 662 keV. It should be noted that a problem of poor detector response in the pulse-height spectra often occurs due to the slow-rising events phenomenon, and that a chemical treatment for surface processing can be applied to solve this problem [30]. Several design parameters were investigated using finite-element analysis (FEA). These design parameters focus on the anode areas, the ratio of screen length to device height, the ratio of anode diameter to device height and the ratio of insulator thickness to relative permittivity [31,32]. The aim of the FEA simulation is to derive a compressed weighting potential toward the anode and thus improving energy resolution.

### 4.2. Pixelate Electrodes

#### Small pixel effect

**Figure 8.**
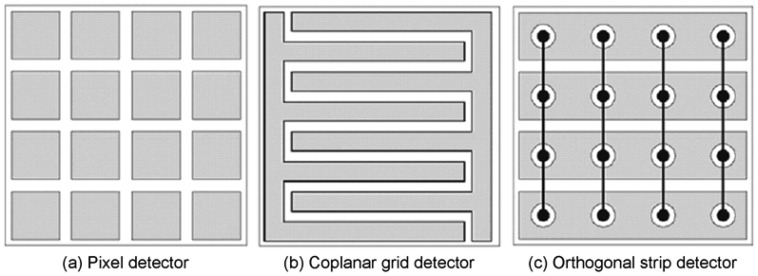
Geometry design of pixelate, coplanar grid and orthogonal strip detector [37] (©IEEE, 2007).

**Figure 9.**
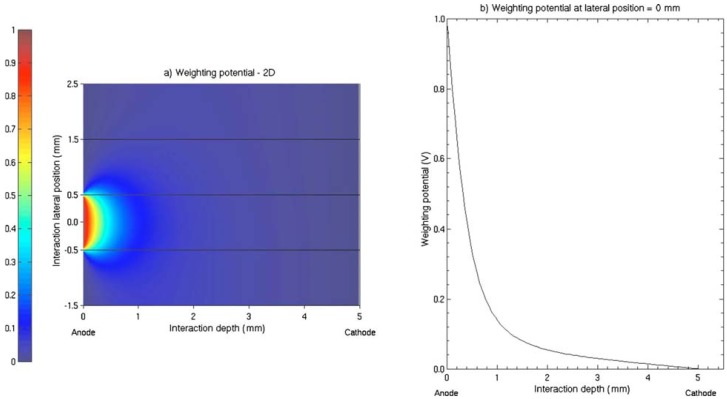
The shape of weighting potential in a pixelate CZT detector with pixel size of 1mm. **Left:** 2-D distribution of the weighting potential. The small pixel effect can be seen clearly in the vicinity of the pixel's anode. **Right:** the correlation between weighting potential and interaction distance from the anode [41] (Image courtesy of American Association of Physicists in Medicine).

Pixelate detectors are the best choice for use in medical imaging since they allow directly for position determination, and semiconductor pixel arrays are more attractive than scintillators in that they can obtain better energy resolution easily [35]. The basic structure of pixel arrays is shown in Figure 8(a). The basis of this design is that charge extraction becomes more localized near the anode as the ratio of width to thickness of the pixel cell (aspect ratio) is decreased. This effect is called “small pixel effect” or “near-field effect”, which is explained in detail by Barrett *et al.* in [36]. The weighting potential distribution is shown in Figure 9.

Commonly, the small-pixel-effect device can deliver good energy resolution with the required corresponding pixel size, and has the advantage of rendering information on spatial interaction locations. Therefore, it allows for operation as an imaging array similar to some scintillation crystal imaging arrays. Experimental results confirm the single charge carrier sensing property of CdZnTe pixelate detectors based on this small pixel effect [38,39]. Among the design considerations are optimal detector thickness [38] and optimal contact geometries [40]. A suitable “aspect ratio” can obtain good energy resolution and detection efficiency; and optimal contact geometry, including pixel size and width of gap and grid, will enhance the charge efficiency on the pixel contacts and reduce charge collection differences resulting from interaction depth variations.

**Figure 10.**
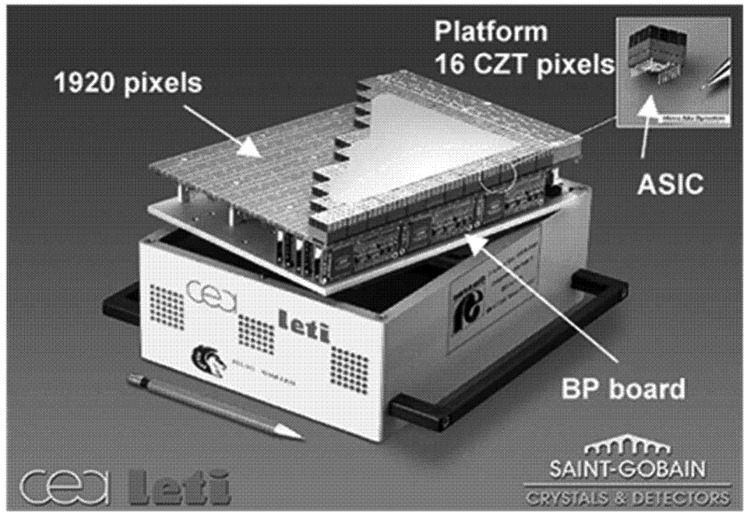
CdZnTe pixel imager prototype by CEA-LETI [42] (©IEEE, 2004).

Recently, prototypes of pixelate detectors were developed by several groups, such as the detectors by Electronic, Technology, and Instrumentation Laboratory, French Atomic Energy Commission (CEA-LETI) [42,43] and University of Arizona (UA) [44,45]. A detector developed by CEA-LETI has a large active area (180 × 215 mm^2^, see Figure 10) and reaches an energy resolution of 4.7% FWHM at 140 keV with BP correction [42]. A semiSPECT detector reported by UA is characteristic of small pixel size (typically 0.38 mm) and high sensitivity. The spatial resolution along the axis is 1.45 mm and the energy resolution is 10% FWHM at 140 keV. There was also a kind of pixelate CdZnTe drift detector reported by Kuvveletli and Budtz-Jørgensen in 2005 [46]. Energy resolution of this drift detector is 3% FWHM at 122 keV with pixels of 0.2 mm dimensions. A commercial micro-SPECT system using a CdZnTe pixelate detector was reported in 2006 [47,48].

**Figure 11.**
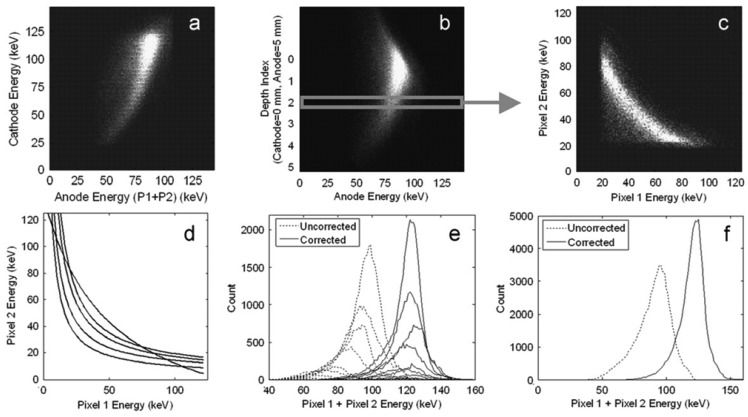
The bi-parameter spectrum (**a**) and the correcting steps for two-pixel charge sharing events. The events plot based on the DOI (**b**) is used to extract different subdivision of pixel-to-pixel plot (**c**). Each subdivision is corresponding to a cluster of events with a certain DOI that is based on the cathode/anode signal ratio. For each subset of events, a correlation curve can be fitted, illustrating the lateral position dependence of charge sharing, thus a family of curves are generated (**d**). Then a table of correction coefficients is obtained and applied to the subsets to correct the energy of each event. Each anode spectrum is shifted after correction (**e**) and an improvement in the charge sharing spectrum is obtained (**f**) [49] (Image courtesy of Elsevier).

One drawback of pixelate detectors is that the pixelate devices suffer from charge sharing problem among pixels. The electronics to solve this problem can be challenging. A data correction method (Figure 11) was reported to correct two-pixel charge sharing events [49]. According to this method, the output signal reduction results from two aspects: induced charge dependence of DOI and the influence of lateral interaction location on charge collection efficiency. The induced charge is dependent on the DOI because of some electron trapping and non-zero weighting potential distribution in the region far away from the anode. The charge inefficiency phenomenon due to the lateral interaction location can be seen in Figure 11(c). The events distribution does not follow a diagonal line as it should have because charge sharing occurs between two adjacent pixels. The sum signal of the two charge sharing pixels between the anode pads has more electron loss. Therefore, both corrections, including a DOI correction and a lateral interaction correction, should be applied together to solve charge sharing problem (Figure 11).

### 4.3. Coplanar-Grid Detectors

The coplanar-grid electrode concept, first reported by Luke [50,51] and based on the principle of Frisch grids, was an innovative single charge sensing method using parallel strip electrodes on the anode. These strip electrodes are connected in an alternate manner to form two banks of grid electrodes (Figure 9(b), here we call them electrode 1 and 2). One set of the grids, electrode 1, are applied by a slightly higher positive voltage than that of electrode 2. Thus the selected charge carriers are always collected by electrode 1. The output signal can be obtained by reading the difference signal between these two sets of electrodes.

**Figure 12.**
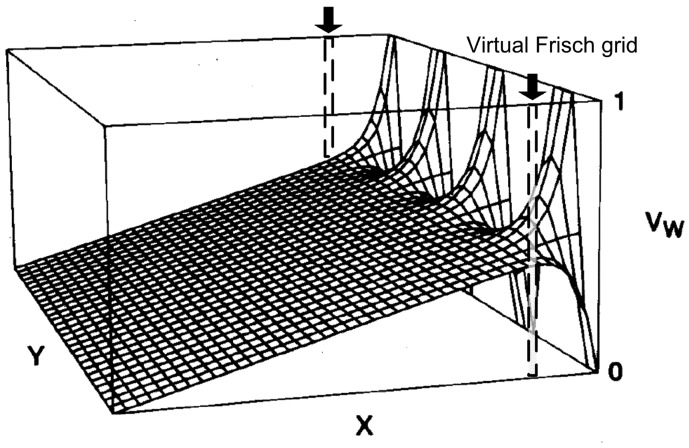
Weighting potential model of one set of coplanar grids. The effect of potential distribution is equivalent to a Frisch grid detector with two parallel strips (in dashline) on side surfaces.

The weighting potential distribution that is obtained using finite element analysis is shown in Figure 12. It is obvious that there is an abrupt variance for weighting potential distribution in the vicinity of anode. Hence, only the carriers drifting to the area near the anode will contribute a lot to the induced charge on the electrode. The weighting potential distribution effect, which is formed in this structure, is equivalent to that formed by two parallel strips on side surfaces (dashed line in Figure 12) on a Frisch grid detector. Therefore, the charge collecting effect is much similar to that of Frisch grid detector. From the perspective of “capacitance coupling method” analysis, the weighting factor *C_a_(x)/C_t_(x)* is equal to *x/(2L)* since the area of collecting electrodes is just one-half of that of the similar planar detector. With this version of detector, an energy resolution of 3.1% FWHM at 662 keV was obtained initially [50,51] and the most promising result was as good as 1.3% FWHM in [52]. However, that result is still much worse than theoretical predicted value of ∼0.3% FWHM.

The key for coplanar grid design is that the weighting potential of the two anode grids are almost equal inside most of the detector volume except the vicinity of the anode. Therefore the subtraction of the two anode grids signal achieves a near-zero weighting potential inside most of the volume. As a result the subtraction signal is only sensitive to the motion of electrons in the vicinity of the anode. Several factors resulting in performance degradation have been studied [4,50,53,54], including electron trapping due to the spatial non-uniformity of the CdZnTe materials and coplanar grid electrode patterns. Luke solved the electron trapping problem that affects the detector performance by reducing the gain of non-collecting grid signals, however, such a compensation method only provides a correction in the circumstance that electron trapping is linear with interaction depth [50,51,53]. A further study by He's group proposed a correction method for electron trapping in the non-linear case using a position sensing technique [52,55]. This technique also shows that better energy resolution can be obtained for those events near cathode than that near anode, whereas electron trapping would be the most severe for events generated near cathode since they drift the longest distance to reach the anode. From this viewpoint, it can be inferred that the primary limiting factor that results in resolution degradation is not the electron trapping. He *et al.* verified this point and identified the problems of non-symmetric effect that causes detector performance degradation. By adding a boundary electrode and then adjusting the strip width of the outermost grids [56], the detector performance was more uniform at different interaction depths.

A drawback of coplanar-grid detector is that it requires two sets of output readout electronics, which inevitably will import more electronic noises. In addition, there is a trade-off between the excessive noise and collection efficiency of the coplanar anodes. The bias voltage should be sufficient to collect enough carriers but cannot reach a very high level before electric noises and leakage currents begin to overwhelm the effective signals.

### 4.4. Hemispherical Electrodes

**Figure 13.**
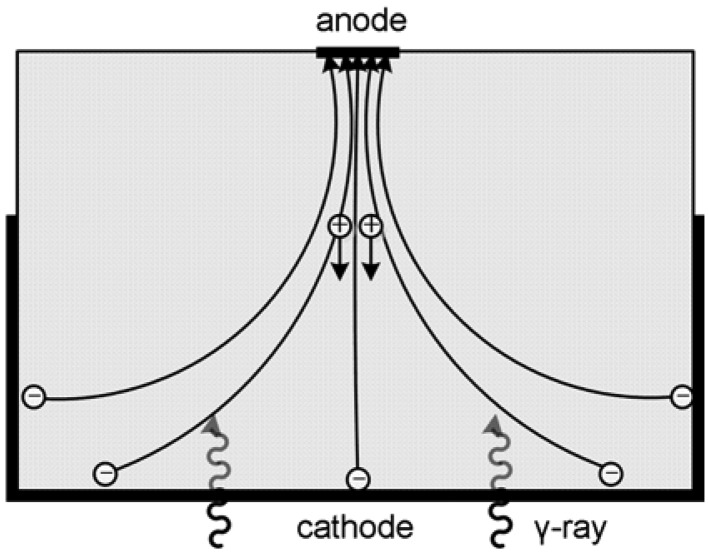
Geometry diagram of hemispherical detector.

The basic concept of hemispherical electrodes is to increase the electric field in the region of the detector where carrier trapping is more frequent, thus attaining a uniform charge collection across the whole area of the detector [57–59]. As shown in Figure 13, this version of electrode is designed with such a shape that there is a small plane anode and a nearly spherical surface as the cathode.

Such a design has two primary effects: one is that a higher electric field near the anode can sweep holes more effectively; the other effect is that more charge carriers are generated near the cathode and the holes concentration near the anode is very small. The combination of these two effects renders the hemispherical electrodes as a single-charge-sensing electrode. With this configuration, energy resolution of 6% FWHM at 662 keV was obtained using a detector with dimensions of 10 × 10 × 5 mm^3^[60]. This kind of detector, which was designed by Parnham *et al.* [61] with a full-area anode and an extended cathode, is commercially available. The dimension of this detector is 5 × 5 × 5 mm^3^ with energy resolution of less than 1.9% FWHM at 662 keV achieved with the optimal configuration.

### 4.5. Strip Electrodes

A research group at the University of New Hampshire Space Science Center developed the earliest prototype of CdZnTe strip detector [62–64]. This device was designed using a monolithic CdZnTe substrate with orthogonal strips on each surface, so that a “pixel” is generated in the overlapping area of two cross orthogonal strips. Position determination can be achieved with this design and good spatial resolution could be obtained with small width strips. Furthermore, a concept of signal analysis was proposed in which the pulse height of neighboring strips can be used to locate individual events more precisely and their sum is a more accurate measurement of gamma ray energy. The drawbacks of this two-sided electrode design are that the signal on cathode, which has a comparatively slow rise-time, still suffers from hole trapping and that it needs electronic readout on both of the two sides.

#### Three-electrode model

**Figure 14.**
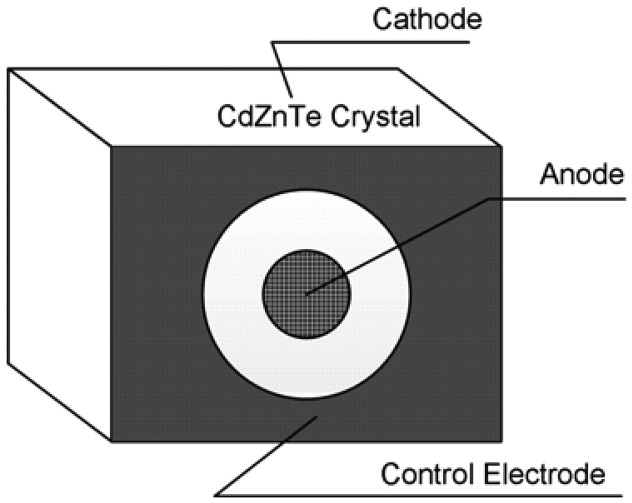
Schematic diagram of three-electrode model gamma ray detector.

**Figure 15.**
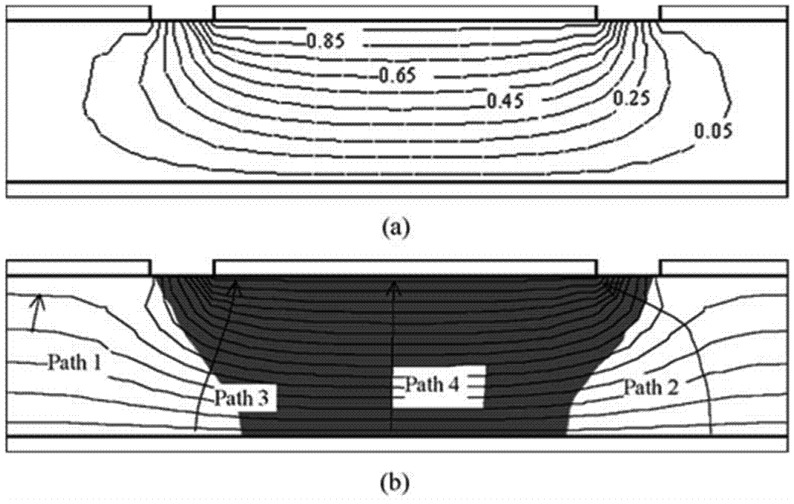
Calculated weighting potential (**a**) and real potential distribution (**b**) of the three-electrode detector. The dark area represents the electric field strength above 2,500 V/cm. The drift path of carriers is determined by the electric field distribution. Charge carriers with a trajectory of path 1 are prone to be trapped due to the weak electric field; path 2 and 3 tend to induce a complete signal on the collecting electrode and path 4 will be similar to that of the planar detector. A commercial simulation program, Quickfield™ by Tera Analysis, was used for both calculations [68] (©IEEE, 2004).

The new concept of “three-electrode model” or “coplanar pixel and control electrode model”, which was reported in 1997 by Lingren *et al.* [65] and Butler [66], aimed at suppressing the long spectrum tail at low energy. This model consists of three electrodes: the cathode, the anode (the collecting electrode) and a control electrode (Figure 14). Many of such models can be connected together to form a gamma ray detector (Figure 8(c)). The control electrode has two functions: one is to assist the anode in focusing most of the charge carriers from the entire volume of the detector; the other function is to reduce electron trapping by shaping the electric field in the detector in such a way that electrons will reach the anode much faster. The real electric field and weighting potential distribution of the three-electrode are shown in Figure 15. Both of the electric field and weighting potentials near the anode are intensive so that the induced charge is independent of interaction position. Therefore, both of the energy resolution and photopeak efficiency can be greatly improved by mitigating the contribution of hole trapping on the output signal. Based on this basic three-electrode idea, a multiple electrode detector model that was equipped with five electrodes was reported in 2007. (more details can be seen in [67].

#### Detector design

**Figure 16.**
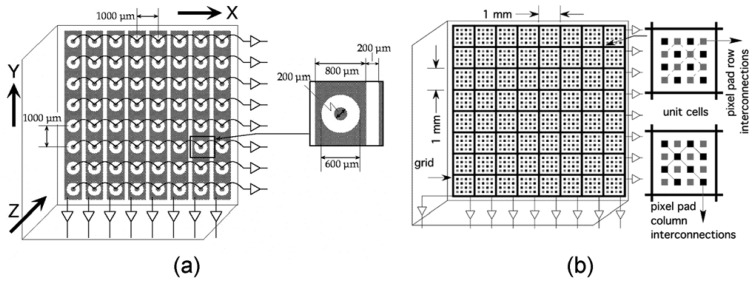
Schematic of the orthogonal coplanar strip detectors (**a**) and the charge-sharing strip detector (**b**). Both of them have a single planar cathode electrode on the opposite side. The anode pixels in (a) and pixel pads in (b) are interconnected in rows to collect the electron charge carriers. The strips in (a) register signals that are induced by electrons as they drift towards to the anode pixels; while the pixel pad columns in (b) are the same as the pixel pad rows but provide signals about their columns information [13,69] (Image courtesy of SPIE and IEEE).

**Figure 17.**
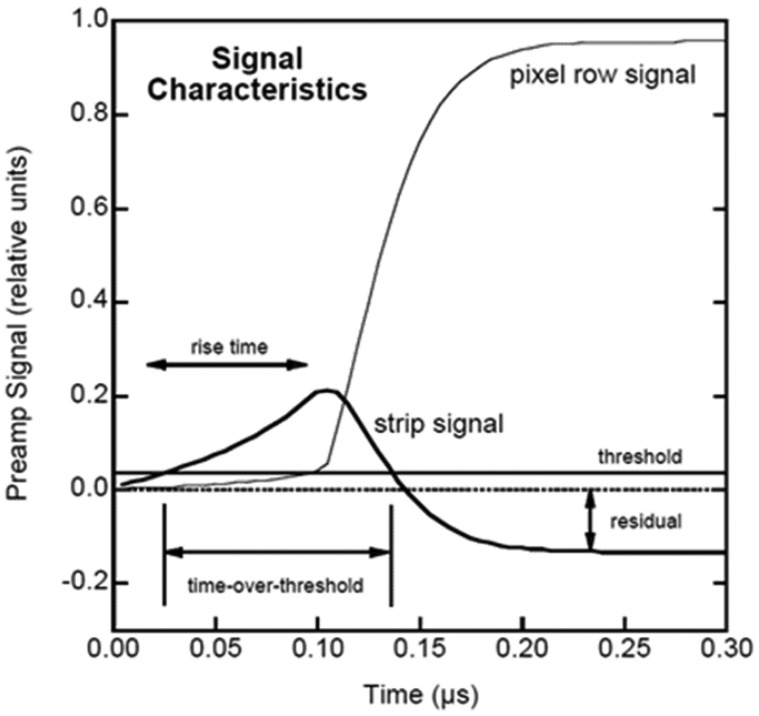
Typical signal shape of the strip and pixel anode signals generated by a single interaction in the orthogonal strip detector. Three features, such as risetime, time-over-threshold and residual value, can be utilized to infer the DOI [69] (Image courtesy of SPIE).

Two kinds of strip detector (Figure 16), an orthogonal coplanar strip detectors [69,70] and a charge-sharing strip detector [13], were developed by the University of New Hampshire and based on the concept of the three-electrode model metioned above. Both of these designs belong to single-sided detector that can contribute N^2^ pixels but only need 2N electronics, which greatly reduce the power requirement and complexity of the device electronics. As for the orthogonal strip detector, a bias voltage difference is required to be applied between the anode and control electrode. This difference value is dependent on its geometric design [68,71]. A higher difference value would result in more leakage current. Typical signals generated in this version detector are shown in Figure 17. X- and Y-coordinates of the event interaction location could be identified by the “strip signal” and “pixel row signal” respectively. Furthermore, the interaction depth (Z-coordinate value) can be inferred qualitatively from the characteristics of the cathode signal or quantitatively from the ratio of the cathode signal to the largest “pixel row signal”. With this design, energy resolution of 1% FWHM at 662 keV was obtained with a 5 mm thick detector [13].

Compared to an orthogonal strip detector, the advantages of the charge-sharing strip detector include: (1) the electronics are simplified due to fact that the row and column electrodes are identical and therefore their output signals are of the same shape; (2) the grid electrodes have a larger area and thus they can provide a more effective non-collecting signal than that provided by the individual strip electrodes in orthogonal strip detector. The disadvantage, however, is that electronic noises are generated from so many pads and also by adding the signal of these pads. Further design details for the two versions of detectors, including the analog processing circuits design [72], edge and corner effect problems [73], and how to balance the pad dimensions and the detector thickness (electron clouds effedt) [13,74–76], can be referred in corresponding references.

Another kind of strip detector concept, which is called drift strip detector, was first introduced in 1987 and developed using silicon material [77]. Then Patt *et al.* [78] applied this design to developing a high-Z compound semiconductor detector using HgI_2_ and fabricated a detector prototype that achieved an energy resolution of 0.9% at 662 keV.

**Figure 18.**
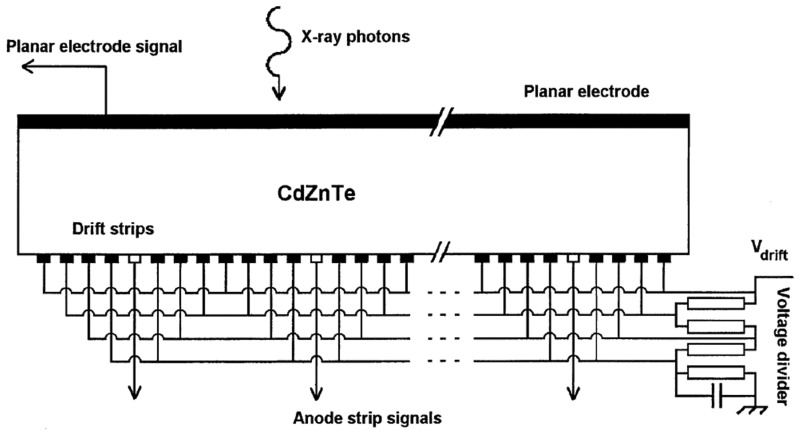
Schematic diagram of CdZnTe drift detector [81]. Such detector has a planar electrode as the cathode, while the anode and drift strips are biased by a voltage divider (Image courtesy of Elsevier).

The first CdZnTe drift detector was developed in the Danish Space Research Institute [79,80] and its schematic diagram is shown in Figure 18. This type of detector is single-sided with a planar electrode on one side and strip electrodes on the other side. An readout anode is in the center surrounded by several drift electrodes, which provide an electrostatic shield to the readout anode so that the sensitivity to hole trapping is reduced. The best energy resolution performance obtained with this detector is 0.8% at 356 keV with spectrum correction for hole trapping [81]. In such a detector, DOI can also be derived by analyzing a parameter R, which is the ratio of the signal on the cathode to that on the readout anode. Compared with a coplanar grid detector, this drift strip detector has simpler electronics since no summing and subtracting circuits are needed.

A summary of the features and performances of CdZnTe detectors with different geometries mentioned above is shown in Table 1. Some studies focus on the effect of different electrode geometries, aiming to compare the performance of them. For example, the performance of detectors with different anode contact geometries was investigated in [82] using the same crystal and same output electronics. The results show that it is the detector with single pixel geometry that presents the best performance for both low- and high-energy gamma ray sources.

## 5. 3-D Position Sensing Technique and Compton Imaging Detector

### Electron trapping problems

Although hole trapping is the key factor in degrading spectrum performance, electron trapping does exist in practice. It was observed experimentally that around 5–10% of electrons that are generated by γ-ray interactions are trapped on a 1 cm thick CdZnTe detector [52]. The electron trapping effect on the signals on electrodes was found in Luke's coplanar grid detector because the subtraction signal is dependent of DOI [51]. Luke solved this problem by reducing the gain of non-collecting grid signal in order to compensate for the electron charge losses. However, this compensation method is based on an assumption that the electron trapping is a linear function of DOI. Therefore, it can only be used on occasions where electron trapping is not too severe and is spatially uniform. A more accurate method for correcting electron trapping in the non-linear case was first reported by He *et al.* [52] using the depth sensing method. The DOI can be inferred by the ratio of the cathode to anode signal and a position resolution of 0.9 mm FWHM at 122 keV was obtained. With electron trapping correction, detector performance would be further improved.

The first prototype of 3-D position sensing spectrometer was developed and introduced by He *et al.* [83,84] based on the 2-D position sensing method and depth sensing technique using a pixelate detector array. This 3-D position sensing technique can be effectively used for correcting material non-uniformity and electron trapping. It also has advantages in analyzing detector response so that the possible defects that significantly degrade the energy resolution can be identified clearly. For example, it was demonstrated experimentally that small lateral size electron-trapping defects do significantly degrade energy resolution of the corresponding pixels, but larger ones do not [85]. Another three generations of spectrometers were subsequently reported in 2004 [86], 2005 [87,88], 2007 [89] and 2012 [90] with different generations of ASIC readout systems respectively.

**Figure 19.**
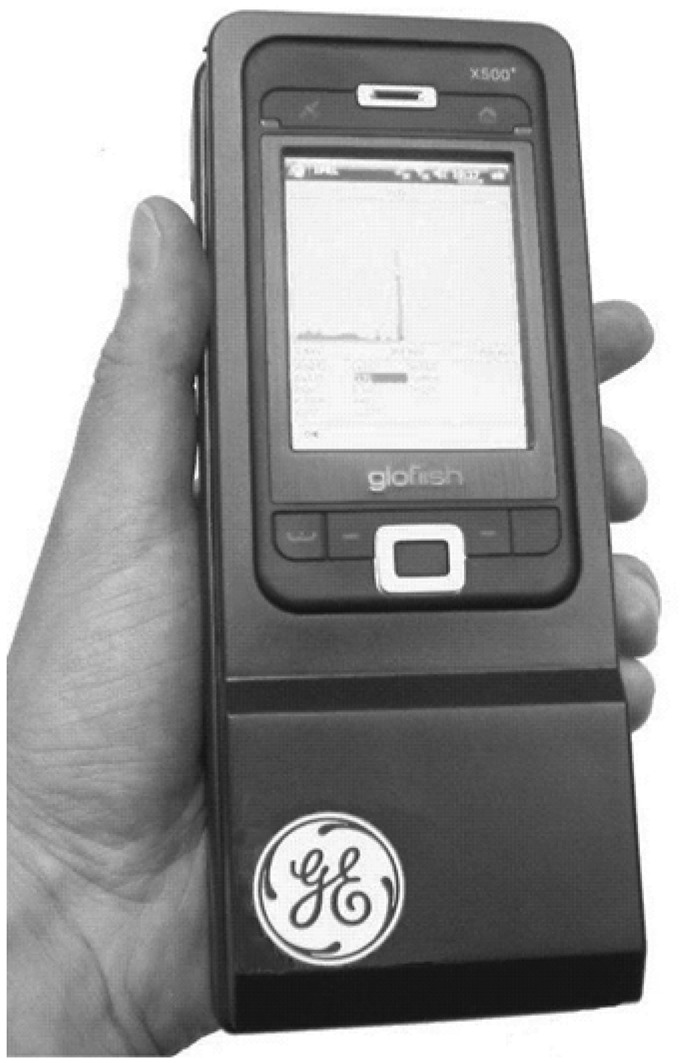
Photo of the GE prototype of IPRL system [94] (Image courtesy of SPIE).

Depth sensing technique in CdZnTe enables Compton imaging [91–93], which can be used to localize the position of radiation source. Compton scatters generated in the CdZnTe detectors will give rise to two gamma-ray interaction events. When both of the energy and position of the two events are measured using 3-D position technique, the direction of the radiation source can be determined by a back projection cone. Compton imaging technique makes it possible to perform intelligent gamma-ray spectroscopy. A typical Intelligent Personal Radiation Locator (IPRL) system was developed and reported by GE researchers [94]. This IPRL detector consists of multiple CdZnTe modules and each module has a CdZnTe crystal with dimensions of 15 × 15 × 10 mm^3^. Having a tatal volume of approximately 700 cm^3^ and weighing under 900 g, the IPRL system (Figure 19) can achieve an energy resolution of 5% FWHM at 122 keV and a location accuracy of less than 4 m.

A corresponding networked system of IPRLs was also designed to improve the abilities of detection, localization, and identification for potential radiological threats [95]. For Compton imaging camera, the angular resolution is a key performance parameter. However, it is limited by the lateral position resolution that is always determined by the pixel pitch dimensions of the detector. A sub-pixel position sensing method was studied by Zhu *et al.* [96] based on non-charge-collecting transient signals [97,98]. The fundamental is that the peak amplitudes of the eight-adjacent neighboring transient signals are dependent of the lateral position of the electron clouds that enter the anode region. This method breaks though the limit of position resolution by pixel pitch of CdZnTe detectors and effectively improves the angular resolution of Compton imaging camera.

## 6. Conclusions

The CdZnTe semiconductor is now regarded as the most promising candidate for the next generation of gamma ray detectors, with the increasing demand for gamma ray imaging devices and significant progress in producing high quality crystals. For imaging devices, CdZnTe semiconductor can obtain good energy resolution easily in comparison with scintillation detectors. Furthermore, spectroscopic performance can be improved effectively by designing special electrode geometry and developing new electronic signal processing techniques. Some technique renders the energy resolution to be greatly improved to below 1% FWHM. However, there is still some room for improvement in real applications, including production of large and uniform crystals, design of very small electrodes (<100 microns), signal processing methods and Application Specific Integrated Circuit (ASIC) with low electronic noise and leakage current. The combination of these techniques will produce a gamma ray detector with good energy resolution and detection efficiency.

## Figures and Tables

**Table 1.** Summary of the features and energy resolution performance of CdZnTe detectors with different geometries.

**Geometry Type**	**Advantages**	**Disadvantages**	**Best Performance in Energy Resolution (FWHM)**
Planar electrode	Simple structure	Severe hole trapping problems	—
Frisch strip and trapezoid prism electrode	Simple structure	Existing leakage currents between the grid and anode	2.68% at 662 keV
Insulated Frisch ring electrode	Eliminating leakage currents between the grid and anode	More complicated design and fabrication technique	1.70% at 662 keV
Pixelate electrode	Higher charge collection efficiency; suitable for medical imaging	Charge sharing problems	<3% at 140 keV
Coplanar grid electrode	Overcoming hole trapping more effectively	Needing more output readout electronics; more electronic noises	1.3% at 662 keV
Hemispherical electrode	Uniform charge collection	Complicated geometry design	<1.9% at 662 keV
Orthogonal coplanar strip electrode	Less complexity for the device electronics	Leakage current in anode	1.0% at 662 keV
charge-sharing strip electrode	Simplified electronics and more effective non-collecting signal	More electronic noise	<6% at 122 keV
Drift strip electrode	The sensitivity to hole trapping is reduced due to the electrostatic shield to the readout anode	More electronic noises	0.8% at 356 keV
